# Shiga toxin-producing Escherichia coli O157:H7 in Japan.

**DOI:** 10.3201/eid0502.990222

**Published:** 1999

**Authors:** J Terajima, H Izumiya, A Wada, K Tamura, H Watanabe


clinical data were unknown. On the same day, we contacted and interviewed the patient to investigate risk factors for cholera. The patient reported abdominal pain starting on 6 June and then severe diarrhea (10 to 12 stools per day) until 13 June; on that day the patient went to his general practitioner, who gave him loperamide and suggested a coproculture. The patient never traveled to cholera-endemic areas; did not eat raw mussels, uncooked fish, or vegetables of uncertain origin or from cholera-infected areas; and did not swim in rivers or lakes. The patient reported that he ate a seafood salad in the canteen of his work place on 5 June and that three out of the four persons who ate the same kind of salad also had abdominal symptoms. Subsequently, the Istituto Superiore di Sanità (ISS) in Rome confirmed isolation of toxinogenic *V. cholerae* O1, biotype El Tor, serotype Ogawa, from stools from the index patient.

The canteen where the index patient had eaten the seafood salad was 1 of 17 supplied by a single cooking center that used a precooked, frozen, ready-to-eat product including shrimps, scallops, mussels, hen clams, cuttlefishes, and squid. Each product was cooked and frozen in the country of origin and mixed in Italy by an importer who packaged the seafood salad. Tracking the products around the world was difficult, but we learned that at least some had come from Far East countries where cholera is endemic. Approximately 125 servings of the same food were distributed within our local public health area (Azienda Sanitaria Locale) and more than 400 in other areas.

We performed an epidemiologic case-control investigation beginning 18 June involving 454 persons (94 who had eaten the seafood salad and 360 controls who had eaten in the same canteen any food except seafood salad); 37 (39%) of the persons who had eaten the seafood salad had had at least one episode of diarrhea or other relevant gastrointestinal symptoms, as compared to one (0.3%) of those who had not eaten it. We did not find symptomatic patients. The corresponding odds ratio was 233 (95% confidence interval, 97 to 560). No symptomatic person had to be hospitalized because of symptoms or required intravenous treatment; three or more loose or watery stools during a 24-hour period were reported in 24 cases. We performed coprocultures (using TCBS medium) between 23 June and 3 July of all 94 persons who had eaten the seafood salad. One positive coproculture for *V. cholerae* O1 Ogawa (same strain) was identified on 25 June; the isolation was subsequently confirmed by the ISS. This second patient with a positive culture worked in a factory in a different town. She had severe diarrhea on 6, 7, and 8 June. Her family doctor gave her rifaximin but did not ask for a coproculture, but a specimen was obtained on 23 June. She did not report risk factors for cholera infection, except having eaten seafood salad on June 5 in the canteen of her work place. The delay between exposure to *V. cholerae* and the coprocultures was longer than 1 week (median delay 26 days, range 19 to 31), and it is therefore not surprising that others who had eaten the seafood salad did not have positive results. Both the culture-positive index case-patient and the woman were recultured three more times; negative results were obtained.

The identification of this cholera outbreak is a sentinel episode confirming (1,2) that, if not adequately monitored, food preparation and distribution can cause serious infectious diseases in industrialized countries.

**Luca Cavalieri d'Oro,*† Elisabetta Merlo,† Eugenio Ariano,† Maria Grazia Silvestri,† Antonio Ceraminiello,† Eva Negri,* and Carlo La Vecchia*‡**

*Istituto di Ricerche Farmacologiche "Mario Negri," Milan, Italy; †Azienda Sanitaria Locale della Provincia di Lodi, Lodi, Italy; and ‡Università degli Studi di Milano, Milan, Italy

### References

1. Centers for Disease Control and Prevention. Cholera associated with food transported from El Salvador—Indiana, 1994. MMWR Morb Mortal Wkly Rep 1995;44:385-6.
2. Centers for Disease Control. Cholera associated with imported frozen coconut milk—Maryland, 1991. MMWR Morb Mortal Wkly Rep 1991;40:844-5.

### Shiga Toxin–Producing *Escherichia coli* O157:H7 in Japan

**To the Editor:** Shiga toxin-producing *Escherichia coli* (STEC) O157:H7 infection, which can cause hemolytic uremic syndrome and death, is a global public health concern. Patients younger than 5 years of age are at high risk for hemolytic uremic syndrome (1) and shed the organism longer than adults (2). The public health importance of this symptomatic shedding in transmission among preschool children is well established (3); however, that of symptom-free shedding in adults is unknown. We report here that the rate of symptom-free STEC O157:H7 shedding is higher in adults 30 to 49 years of age than in others.

STEC infections have been notifiable in Japan since August 1996. When STEC is found in the feces of patients in schools, families, and hospitals, local health centers and public health institutes must test (generally using MacConkey sorbitol agar with cefixime-potassium tellurite medium) for the pathogen in stool specimens of contacts of the patients. The pathogen is also sought twice a month in the stool specimens of food-handlers. All isolates from culture-positive patients are collected by Japan's National Institute of Infectious Diseases.

In 1997, 1,412 STEC O157:H7 human isolates were examined for subtyping of Shiga toxin genes *stx1* and *stx2* by polymerase chain reaction, for genotyping by *Xba*I-digested pulsed-field gel electrophoresis (PFGE) (4,5), and for their relationship with symptoms; 1,381 isolates (from culture-positive persons with well-characterized clinical status) were further analyzed. The rates by age group among STEC O157:H7-shedding persons reporting one or more symptoms (vs. culture-positive persons without symptoms) were as follows: 82% (475 of 576) younger than 10 years old; 81% (145 of 178), 10 to 19 years; 63% (98 of 156), 20 to 29 years; 25% (32 of 128), 30 to 39 years; 34% (34 of 100), 40 to 49 years; 54% (57 of 106), 50 to 59 years; 56% (38 of 68), 60 to 69 years; 68% (47 of 69), older than 70 years. Culture-positive persons under 20 years of age, especially children under 10 years of age, were more likely to have symptoms than other age groups. Intermediate rates of symptom-free persons with positive stool cultures occurred in young adults (20 to 29 years of age) and the elderly (70 years of age), while the highest rates of stool-positive, symptom-free persons were adults, especially those between 30 and 49 years of age. In terms of pathogen virulence, we did not find significant differences in the distribution of *stx* subtypes and PFGE genotypes between strains shed by symptomatic and asymptomatic persons. These results suggest that the rate of symptom-free STEC O157:H7 shedding may be associated with age rather than organism-related factors. Possible age-related host factors that could influence the presence of STEC O157:H7 in the stools of symptom-free persons include qualitative and quantitative differences in intestinal cross-reactive antibodies against STEC O157:H7, intestinal bacterial flora, or the sensitivity to Stx toxins between children and adults. Further investigations will be required to determine the relative importance of these and other host factors. Our finding of a high rate of asymptomatic shedding in adults may suggest the potential for secondary transmission of the bacteria from symptom-free STEC O157:H7-shedding adults to healthy children.

**Acknowledgments**

We thank investigators of prefectural and municipal public health institutes for their collaboration.

This work was supported by a grant from the Ministry of Health and Welfare of Japan.

**Jun Terajima, Hidemasa Izumiya, Akihito Wada, Kazumichi Tamura, and Haruo Watanabe**
National Institute of Infectious Diseases, Tokyo, Japan

**References**

1. Tarr PI. *Escherichia coli* O157:H7: clinical, diagnostic, and epidemiological aspects of human infection. Clin Infect Dis 1995;20:1-8.
2. Belongia EA, Osterholm MT, Soler JT, Ammend DA, Braun JE, MacDonald KL. Transmission of *Escherichia coli* O157:H7 infection in Minnesota child day-care facilities. JAMA 1993;269:883-8.
3. Karch H, Russman H, Schmidt H, Schwarzkopf A, Heesmann J. Long-term shedding and clonal turnover of enterohemorrhagic *Escherichia coli* O157:H7 in diarrheal diseases. J Clin Microbiol 1995;33:1602-5.
4. Watanabe H, Wada A, Inagaki Y, Itoh K, Tamura K. Outbreaks of enterohaemorrhagic *Escherichia coli* O157:H7 infection by two different genotype strains in Japan. Lancet 1996;348:831-2.
5. Izumiya H, Terajima J, Wada A, Inagaki Y, Itoh K, Tamura K, et al. Molecular typing of enterohemorrhagic *Escherichia coli* O157:H7 isolates in Japan by using pulsed-field gel electrophoresis. J Clin Microbiol 1997;35:1675-80.

### *Streptococcus pyogenes* Erythromycin Resistance in Italy

**To the Editor:** *Streptococcus pyogenes* resistance to erythromycin began to emerge as a serious problem worldwide in the early 1990s. In some areas in Italy, 30% to 40% of strains have beome

